# Nanostructured surfaces for analysis of anticancer drug and cell diagnosis based on electrochemical and SERS tools

**DOI:** 10.1186/s40580-018-0143-4

**Published:** 2018-04-24

**Authors:** Waleed A. El-Said, Jinho Yoon, Jeong-Woo Choi

**Affiliations:** 1 Department of Chemical and Biomolecular Engineering, Sogang University, 35 Baekbeom-Ro, Mapo-Gu, Seoul, 04375 Republic of Korea; 2 Department of Chemistry, Faculty of Science, Assiut University, Assiut, 71516 Egypt

**Keywords:** Electrochemistry, Raman spectroscopy, Anticancer drugs, Drug metabolism, Tumor investigation, Cell-based chip, Surface-enhanced Raman spectroscopy

## Abstract

Discovering new anticancer drugs and screening their efficacy requires a huge amount of resources and time-consuming processes. The development of fast, sensitive, and nondestructive methods for the in vitro and in vivo detection of anticancer drugs’ effects and action mechanisms have been done to reduce the time and resources required to discover new anticancer drugs. For the in vitro and in vivo detection of the efficiency, distribution, and action mechanism of anticancer drugs, the applications of electrochemical techniques such as electrochemical cell chips and optical techniques such as surface-enhanced Raman spectroscopy (SERS) have been developed based on the nanostructured surface. Research focused on electrochemical cell chips and the SERS technique have been reviewed here; electrochemical cell chips based on nanostructured surfaces have been developed for the in vitro detection of cell viability and the evaluation of the effects of anticancer drugs, which showed the high capability to evaluate the cytotoxic effects of several chemicals at low concentrations. SERS technique based on the nanostructured surface have been used as label-free, simple, and nondestructive techniques for the in vitro and in vivo monitoring of the distribution, mechanism, and metabolism of different anticancer drugs at the cellular level. The use of electrochemical cell chips and the SERS technique based on the nanostructured surface should be good tools to detect the effects and action mechanisms of anticancer drugs.

## Introduction

Nanomaterials have been widely used in different applications such as cancer diagnoses, cancer treatments based on drug delivery or photothermal therapy, and the development of highly sensitive and selective sensors for monitoring anticancer drugs effects and their metabolism [1–6]. Studying drugs’ cellular uptake, intracellular distribution, and intracellular interaction with target molecules at the single-cell level (the most fundamental units at which drugs take effect) are important issues for the development of new anticancer drugs. One critical challenge for drug discovery is that the evaluation of a drug’s toxicity is very time-consuming and expensive [7–9]. Currently, many in vitro tools including western blotting, MTT (3-(4,5-dimethylthiazol-2-yl)-2,5-diphenyltetrazolium bromide) assay, apoptosis enzyme-linked immunosorbent (ELISA) assay, spectrophotometric methods, fluorescent microscopy and confocal microscopy [10–14] have been established to study the efficiency of drugs or toxins, perform toxicity analysis with different chemicals, cell proliferation, cell metabolic changes, and discover new anticancer drugs [15–18]. Although these assays have shown reliable and reproducible results, complicated sampling procedures were required, they frequently involved cell destruction, and the obtained data was acquired at a specific time point (end-points) [19, 20]. The disadvantage of many organic fluorescent dyes is their propensity to undergo photobleaching, spectral overlapping, and bio autofluorescence interference; in addition, these labels could change the drugs’ biological distributions and physiological behaviors. Therefore, the development of a noninvasive and high-throughput analytical method is needed for evaluating the potency and efficacy of drugs in vitro during the early stages of drug discovery. Recently, optical and electrochemical cell-based chips have potentially been applied as label-free, in situ, and noninvasive in vitro tools for drug discovery and to analyze the effects of anticancer drugs [21–23]. One important direction of the development of cell-based chips is the adhesion of living cells and cell-to-cell interactions, which could be a reliable candidate for the cellular attachment without the loss of cell viability [24]. Several recent electrochemical cell-based chip techniques have been reported for detecting cell viability and estimating the effects of anticancer drugs without the need for fluorescence dyes or other label agents that could overcome the limitations of traditional assays [25–28]. Electrochemical detection techniques have unique advantages including fast responses, high sensitivity, real-time monitoring, cost-effectiveness, and noninvasiveness.

The principle of these electrochemical cell-based chips was based on recording the electrochemical behavior of the cell’s suspension or confluent cell monolayers on the chip’s surface. In addition, their applications for the discovery of new anticancer drugs by monitoring the changes in cell behavior that are induced by anticancer drugs were based on the results that change in the electrochemical response of treated cells [29–31]. Different electrochemical techniques were used, including impedance spectroscopy (EIS) [15, 17], amperometry, electric cell-substrate impedance sensing (ECIS) [32, 33], cyclic voltammetry (CV) [16, 34–38], differential pulse voltammetry (DPV) [39, 40], open circuit potential at the cell/sensor interface [30], and scanning electrochemical microscopy (SECM) [27, 41, 42]. Raman spectroscopy is one of the most promising label-free rapid and nondestructive techniques for cancer diagnosis, in situ monitoring of the effects, action mechanisms, and distribution and metabolism of different drugs at the cellular level without any sample preparations, which could reduce the need for animal experiments. The Raman phenomenon results from an inelastic scattering of photons by the molecule and it provides information about their chemical composition. Accordingly, nanostructured surfaces could provide highly sensitive electrodes that could be used in the development of electrochemical cell-based chips, to investigate the effect of different anticancer drugs, and for drug discovery. The use of nanostructure-modified electrodes enables the detection of the effects of very-low-concentration anticancer drugs. However, this type of analytical tool cannot help us understand either the drug’s action or mechanism. Furthermore, nanostructured surfaces could be used as a SERS-active substrate that could be applied as a nondestructive tool to study and understand the mechanisms of different anticancer drugs, but the uniform distribution of nanostructures over a large surface area plays a vital role in developing quantitative or semi-quantitative tools for monitoring the effects of anticancer drugs.

## Electrochemical cell chip

### Electrochemical cell biosensor based on a modified carbon electrode

**Fig. 1:**
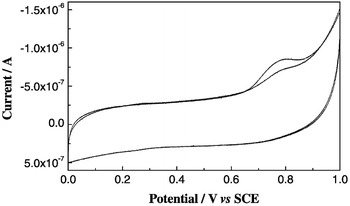
Voltammetric behavior of Hela cells, the scan rate was 30 mV s^−1^, the temperature was 37 ± 0.1 °C, and the cell number was 7.3 × 10^5^ mL^−1^ (Reprinted with permission from [45]. Copyright @ 2001 Elsevier)

Several modified carbon electrodes have been reported for monitoring the cell viability and for investigating the effects of different anticancer drugs on cell functions [43–49]. Feng et al. reported on the uses of graphite as a working electrode for studying the voltammetric behavior of human mammalian cells (human leukemia cells U937, human leukemia cells HL60, and human erythroleukemia cells HEL). They used phosphate buffer solution (PBS, pH 7.4) for cell suspension to study these cell lines’ electrochemical behavior. The results demonstrated that the first scan showed irreversible behavior with an only anodic peak at 640 mV for U937 and HL60 cells lines and at about 560 mV for HEL cells. They mentioned that the electrochemical responses of these cells could be related to the presence of certain enzymes [43, 45]. Furthermore, they applied the CV technique to assess the anticancer activity of caffeic acid and 5-fluorouracil (5-FU) as anticancer drugs on tumor cells, which showed a decrease in the CV response of the cells, which indicated the applicability of the CV method for evaluating the cytotoxicity activities of anticancer drugs [43, 45]. Ci et al. [45] reported the CV behavior of a suspension of HeLa cells in PBS using a graphite electrode as a working electrode, which revealed irreversible behavior with an anodic peak current at about that appeared at 770 mV versus SCE on the first scan and no cathodic peak current, as shown in Fig. 1. They investigated the cytotoxicity effect of the cisplatin drug on cell viability, based on the changes of the oxidation peak current. Chen et al. [46] reported the introduction of multiwall carbon nanotubes modified glassy carbon electrode (GCE) for monitoring cell viability and the effects of antitumor drugs on tumor cells (Leukemia K562 cells) depending on the changes of electrochemical responses. The voltammetric behavior of suspended leukemia K562 cells in PBS exhibited an anodic peak at about + 823 mV. They also monitored the effects of the 5-FU anticancer drug and several clinical antitumor drugs (vincristine, adriamycin, and mitomycin C); the electrochemical responses of treated K562 cells were decreased significantly compared to the results of control cells [46]. Du’s group [47] studied the electrochemical behavior of a monolayer of living pancreatic adenocarcinoma cells (AsPC-1 cells) using colloidal gold nanoparticles (Au NPs) and a modified carbon paste electrode (Au-CPE) that exhibited an irreversible voltammetric response with an anodic peak at about + 813 mV in the first scan and no reduction peak. Moreover, they studied the effect of adriamycin as an antitumor drug on AsPC-1 cell viability based on the difference in the peak current. Their data displayed a drastic decrease in the anodic peak compared with the control, and this decrease grew larger as the treatment period was incremented or the concentration of Adriamycin increased [47]. Hao et al. [48] developed an electrochemical immunosensor composed of a P-glycoprotein antibody modified epoxysilane monolayer modified glassy carbon electrode to study the proliferation of leukemia K562A cells based on the change of the electron-transfer impedance and the voltammetric response of the electrochemical probe. They studied the morphology of the epoxysilane monolayer and bound antibodies using atomic force microscopy, and they mentioned that the electrochemical response was linearly dependent on the number of cells. In these reports, they monitored cell viability and anticancer drug activities of the cell suspension and immobilized cells; however, they suggested that these electrochemical responses might be related to the oxidation of guanine [45–48].

Recently, Zhu and his group (2016) have prepared a purified-multiwall carbon nanotube (p-MWCNT) and ionic liquid modified GCE (p-MWCNT/IL/GCE) for detecting the electrochemical response of HeLa cells, which showed two anodic peaks at + 720 and + 1034 mV. The cytotoxicity effects of the three chlorophenols (2,4-dichlorophenol, pentachlorophenol, and 2,4,6-trichlorophenol) were evaluated based on the changes in the cells’ electrochemical responses [49]. In contrast to previous studies that use carbon modified electrodes, Zhu et al. mentioned that the electrochemical response of the HeLa cells was related to the oxidation of adenine and hypoxanthine [49].

Based on the results of the studies above, it was noted that the electrochemical responses of different cell lines using different modified carbon electrodes have demonstrated irreversible behavior with an anodic peak current at the first scan and no cathodic peak current, and this anodic peak had disappeared in the second scan cycle, which indicated the instability of the cells’ electrochemical responses at the modified carbon electrodes, which could result in inaccurate results. Furthermore, the irreversible responses of the cells led these studies to suggest that the electrochemical responses were related to the oxidation of guanine or adenine. However, it is difficult to relate the electrochemical response of the whole cell for only the oxidation of guanine or adenine (components of DNA), while the cells contain many electrochemically active species including proteins, enzymes, and carbohydrates. Therefore, more research is needed to clarify the cells’ electrochemical responses. The next section focuses on the uses of metal electrodes, which have faster electron-transfer rates compared to carbon electrodes.

### Electrochemical cell biosensors based on modified metal electrodes

**Fig. 2:**
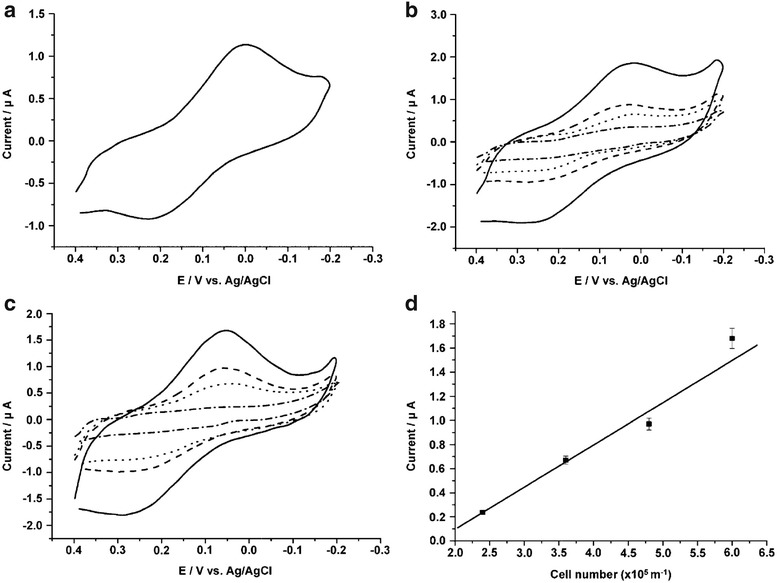
Electrochemical response (**a**) of HeLa cells using a scan rate of 100 mV s^−1^, temperature of 37 ± 0.5 °C, and cell number of 5.8 × 10^5^ mL. **b** Cyclic voltammograms of HeLa cells at different scan rates (dash dot dash lines) 25 m V s^−1^, (dotted lines) 50 m V s^−1^, (dashed lines) 100 m V s^−1^, (solid lines) 200 m V s^−1^. **c** Relationship of peak current with the cell number (dashed lines) 2.4 × 10^5^ cells mL^−1^, (dotted lines) 3.6 × 10^5^ cells mL^−1^, (dashed lines) 4.8 × 10^5^ cells mL^−1^, (plain curves) 6 × 10^5^ cells mL^−1^. **d** Linear plot of reduction current peak as a function of cell number. The scan rate was 100 mV s^−1^ and the temperature was 37 ± 0.5 °C. Data are the mean ± standard deviation of three different experiments (Reprinted with permission from [16]. Copyright @ 2009 Elsevier)

Au electrodes potentially display very favorable kinetic electron-transfer with large anodic potential range [50]. El-Said et al. [16] reported direct HeLa cell immobilization onto Au-patterned silicon electrode as a cell-based chip for cancer diagnosis, drug detection, and the on-site monitoring of the cytotoxicity activity of different anticancer drugs, including hydroxyurea (HU) and cyclophosphamide. The cell growth, cell viability, and cytotoxicity activities were investigated based on CV and potentiometric stripping techniques. Unlike carbon electrodes, adhering HeLa cells onto the Au electrode displayed quasi-reversibility with an anodic peak at + 288 mV and a cathodic peak at about + 60 mV, as shown in Fig. 2. These results illustrated the advantage of using an Au electrode to enhance the electron-transfer rate compared to non-metal electrodes. They mentioned that the voltammetric response of HeLa cells could be related to the presence of some extracted redox enzymes including NADH dehydrogenase (ubiquinone) flavoprotein 2, quinone oxidoreductase-like (QOH-1), and they validated extracting these enzymes from living cells using a 2D electrophoresis technique [16]. The cell viability was monitored based on the change of the cathodic peak current, which displayed a linear relationship as the number of viable adhered HeLa cells on the Au electrode increased. Then, HeLa cells were treated with different concentrations of HU and cyclophosphamide, which induced a decrease in the cathodic current peak and thus decreased cell viability as the dose of the anticancer drugs increased. The CV results were confirmed using Trypan Blue dyeing assay.

Many factors could affect the cell adhesion mechanisms such as the specific surface chemistry, surface hydrophobicity, topography, surface charge, and protein interactions [51]. Strong cell attachment is needed to enhance cell growth over a used chip; therefore, several materials have been used including extracellular matrix (ECM) proteins [52], elastin, and collagen [53] to enhance the cells’ attachment. However, they are quite expensive and could lead to decreased cell chip sensitivity due to the resistance increasing in the interface between the cell membrane and the used chip. Choi and his group reported on the fabrication of a cell-based chip, based on coating Au electrodes with RGD (Arg-Gly-Asp) peptide terminated with cysteine residues to enhance the adhesion of HepG2 cells and the electrochemical sensitivity of the developed cell-based chip [54–58]. A monolayer of HepG2 cells on the RGD oligopeptide-modified surface of the chip was used to assess the anticancer drug activity based on the CV response, which showed a linear increase as the cell number was incremented. These results indicated the capability of RGD peptides to enhance cell immobilization and their voltammetric signal and hence enhance the performance of the constructed cell chip for cancer diagnosis and on-site drug detection.

**Fig. 3:**
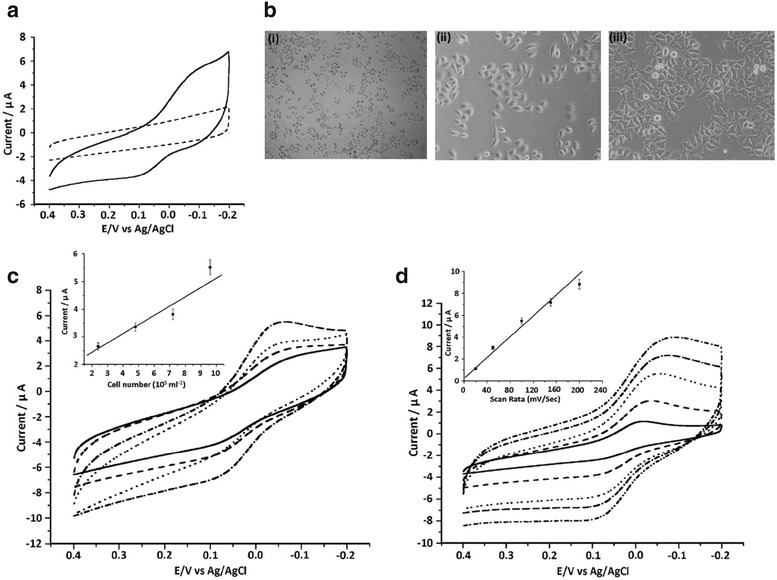
**a** CV for HeLa cell on (dashed lines) ITO and (plain curves) thin film of PANI/ITO, cell number was 5.8 × 10^5^ mL^−1^. **b** Microscopy image of HeLa on (i) ITO incubated for 48 h, (ii) thin film of PANI/ITO incubated for 24 h and (iii) thin film of PANI/ITO incubated for 48 h. **c** CV for HeLa cells on PANI/ITO at different cell numbers, (plain curves) 2.4 × 10^5^, (dashed lines) 4.8 × 10^5^, (dotted lines) 7.2 × 10^5^, (dash dot dash lines) 9.6 × 10^5^. Inset linear plot of reduction current peak as a function of cell number. The scan rate was 0.1 V s^−1^ and the temperature was 37 ± 0.5 °C. **d** CV of HeLa cells on PANI/ITO at different scan rate (plain curves) 20, (dashed lines) 50, (dotted lines) 100, (dash dot dash lines) 150 and (dash dot dot dash lines) 200 mV/s. Inset linear plot of reduction current peak as a function of scan rat. Data is shown as the mean ± standard deviation of three different experiments (Reprinted with permission from [36]. Copyright @ 2009 Elsevier)

Polyaniline (PANI) is a promising and widely used conductive polymer due to its easy synthesis, biocompatibility, and environmental stability [59, 60]. However, the conductive polymers could only show their electrochemical activity in an acidic medium, which restricted their biological applications. Therefore, many efforts have been reported on adapting PANI to be active at a neutral pH by introducing acidic groups into PANI chains [61] or by doping PANI with negatively charged polyelectrolytes [62], but these could affect cell viability. El-Said et al. reported the fabrication of a PANI-based ultrathin film modified indium–tin oxide (ITO) electrode at neutral pH without the co-deposition of an acidic counterion, and its applications as a cell-based chip for cancer diagnosis and for studying the effects of anticancer drugs on HeLa cancer cells [36]. The CV of PANI/ITO in PBS buffer (pH 7.0) represented a cathodic peak at − 73 mV and an anodic peak at + 360 mV. The electrochemical activity of the PANI/ITO electrode at pH 7 was related to the presence of acidic groups on the ITO surface that could act as acidic counterions [63]. The different condition-related parameters (deposition time and the pH of measurements) were then optimized; the results demonstrated that the electrochemical activity was decreased as the pH was increased, which might be related to the nature of PANI that contains both oxidized units (quinoids) and reduced units (benzenoids) [64]. The developed sensor was shown to be a good surface for HeLa cells growth without any significant morphological changes (Fig. 3). Cell growth, cell viability, and anticancer drugs’ efficiency were evaluated by the CV technique of the adhered HeLa cells in PBS buffer (pH 7). In addition, the prepared electrochemical sensor showed high capability to determine the effectiveness of HU and cyclophosphamide as anticancer drugs on HeLa cancer cell viability for a wide range of concentrations [36]. Adlam and Woolley developed a multi-well biosensor composed of eight culture wells (each well containing Au as a working electrode and Ag/AgCl as a reference electrode) for monitoring the viability of HepG2 and rheumatoid synovial fibroblasts cells and the effects of other chemicals on the cell viability based on the changes of the open circuit potential (OCP) [65].

**Fig. 4:**
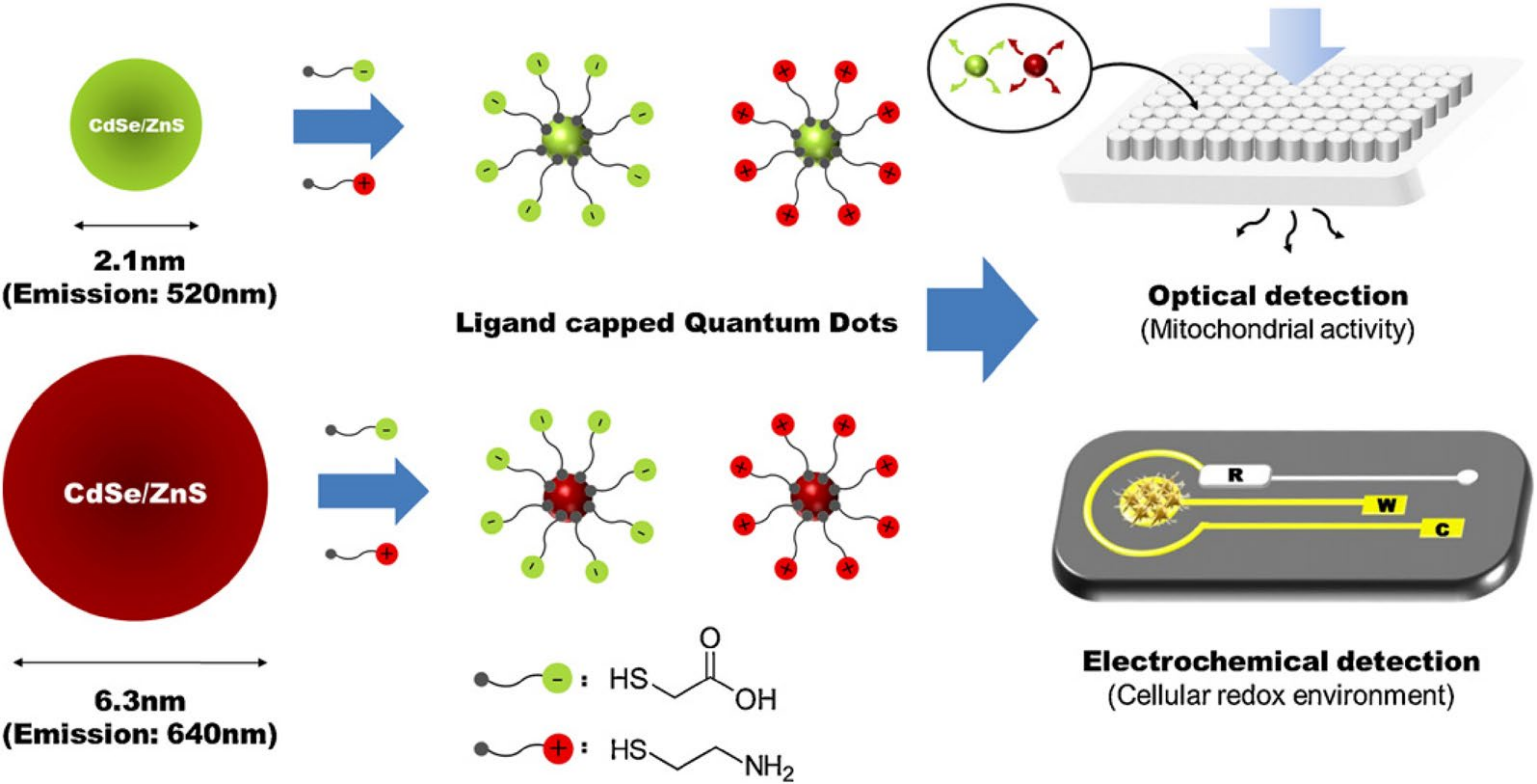
Schematics for the detection of cytotoxicity of thioglycolic acid (TA) or cysteamine (CA)-capped green and red quantum dots (QDs) based on cell chip and conventional MTT assay. ‘R’, ‘W’, and ‘C’ mean the reference, working, and counter electrodes, respectively (Reprinted with permission from [40]. Copyright @ 2012 Elsevier)

The absence of a zinc sulfide shell in single-core quantum dots (QDs) (e.g. CdSe or CdTe) increased the cytotoxic effect of QDs on different cell lines [66], which could be decreased by capping with different ligands [67]. Therefore, there is a need for an intensive assessment of the QDs’ cytotoxic effects on different cell lines. Currently, the cytotoxicity of QDs is determined based on colorimetric or fluorescence-based methods such as MTT viability assay, fluorescence-activated cell sorting assay, and fluorescence imaging [68–70]. However, the natural strong fluorescence of QDs could interfere with signals during the determination of cell viability and create inaccuracies in the obtained data. Choi et al. reported on the fabrication of an electrochemical cell-based chip for monitoring the cytotoxic effects of CdSe/ZnS QDs of two different sizes (green QDs were 2.1 nm and red QDs were 6.3 nm in diameter) on the viability of human neuroblastoma (SH-SY5Y) cells at very low concentrations (Fig. 4). The cytotoxicity effects of green and red QDs capped with cysteamine (CA) or thioglycolic acid (TA) on the SH-SY5Y cell line were investigated using trypan blue and DPV techniques. The cellular uptakes of different sizes of QDs with negative or positive surface charges were confirmed by fluorescence microscopy, and their cytotoxic effects were determined by DPV, MTT, and trypan blue assays. The electrochemical behavior of SH-SY5Y cells immobilized on an RGD-MAP-C peptide-modified Au electrode demonstrated quasi-reversible behavior with an anodic peak at about 365 mV and a cathodic peak at 250 mV. The DPV was used to study the toxicity of 100 μg mL^−1^ of a different kind of TA/CA-capped green or red QD on SH-SY5Y cells, which demonstrated that using TA to cap QDs results in the highest cytotoxicity effects. Moreover, the cytotoxicity effects of different concentrations of TA-capped red/green QDs was monitored by DPV, trypan blue, and MTT techniques. MTT and DPV techniques showed that the cell viability was decreased by 15, 37.5, 11.9, and 39.2% after cells were treated with 5–50 μg mL^−1^ of TA-red QDs and 5–30 μg mL^−1^ of TA-green QDs, respectively. Therefore, DPV is more sensitive than trypan blue and MTT assays for detecting the cytotoxicity of QDs and capped QDs, particularly at low concentrations (1–10 μg mL^−1^) [40]. Ko et al. reported the development of a cell chip composed of a thiolated chitosan monolayer modified Au electrode that enhanced cell adhesion and electrochemical signals [71]. This cell chip was applied to distinguish between normal (HMEC) and breast cancer (MCF-7) cells of the same origin; in addition, this cell chip was successfully applied to screen the effects of two anti-cancer drugs (doxorubicin and cyclophosphamide) based on a CV technique. The CV results were validated based on a conventional MTT assay; hence, this cell-based chip could be used to discriminate normal cells from cancer cells, evaluate the efficiency of newly developed drugs, and assess the cytotoxic effects of doxorubicin and cyclophosphamide as anticancer drugs.

Based on the above-reported data, the electrochemical cell-based chips are quicker, easier, and more sensitive than optical methods for differentiation between cancerous and healthy cell lines, the assessment of cell viability, and the efficiency of several kinds of anticancer drugs and environmental toxins. Although electrochemical cell-based chips showed superior performance for cancer diagnosis and for the assessment of cell viability, they did not allow us to study the action mechanism of anticancer drugs or monitor the metabolism of drugs since the principle of these electrochemical cell-based chips is the changes in the electrochemical response, which depends on the change of electron transfer between the cells and the electrode surface. Therefore, there is a need for a nondestructive method to monitor the metabolism of anticancer drugs in real time and to understand their action mechanisms.

## In vitro SERS technique based on a nanostructured surface to monitor the effects of anticancer drugs and cell metabolism

Raman spectroscopy is a powerful, rapid, label-free, nondestructive analytical technique for in vitro and in vivo analysis [72]. The fundamental principle of Raman spectroscopy is based on the inelastic scattering of photons from targeting molecules that are activated by a laser source. Thus, the real-time monitoring of changes in the biochemical composition of a complicated system such as living cells could be analyzed by examining each peak from the Raman spectra, which is not available with optical, biological, or electrical methods [73]. Raman spectroscopy and surface-enhanced Raman spectroscopy (SERS) were recently reported as powerful techniques for analyzing the chemical composition of a single living cell and for studying the effects of different anticancer drugs on cell viability [74–80]. In addition, SERS showed high capability for the noninvasive investigation of the action of different drug mechanisms because different drugs will have different effects on living cells and further induce changes in biochemical composition. Furthermore, SERS offers an ultrasensitive tool for interfacial studies due to the different Raman signatures and high-throughput screening of various molecules with narrow bandwidths, avoiding spectral overlaps along with the optical interference effects by appropriate nanostructures [80, 81]. In addition, Raman microscopy has recently shown a unique contribution for in vitro and in vivo monitoring of drug release, distribution, and metabolism [82–86], that should impact the therapeutic efficacy and cytotoxicity of drug delivery systems [86].

### In vitro SERS technique for monitoring the effects of anticancer drugs based on peptide/metal nanoparticles

**Fig. 5:**
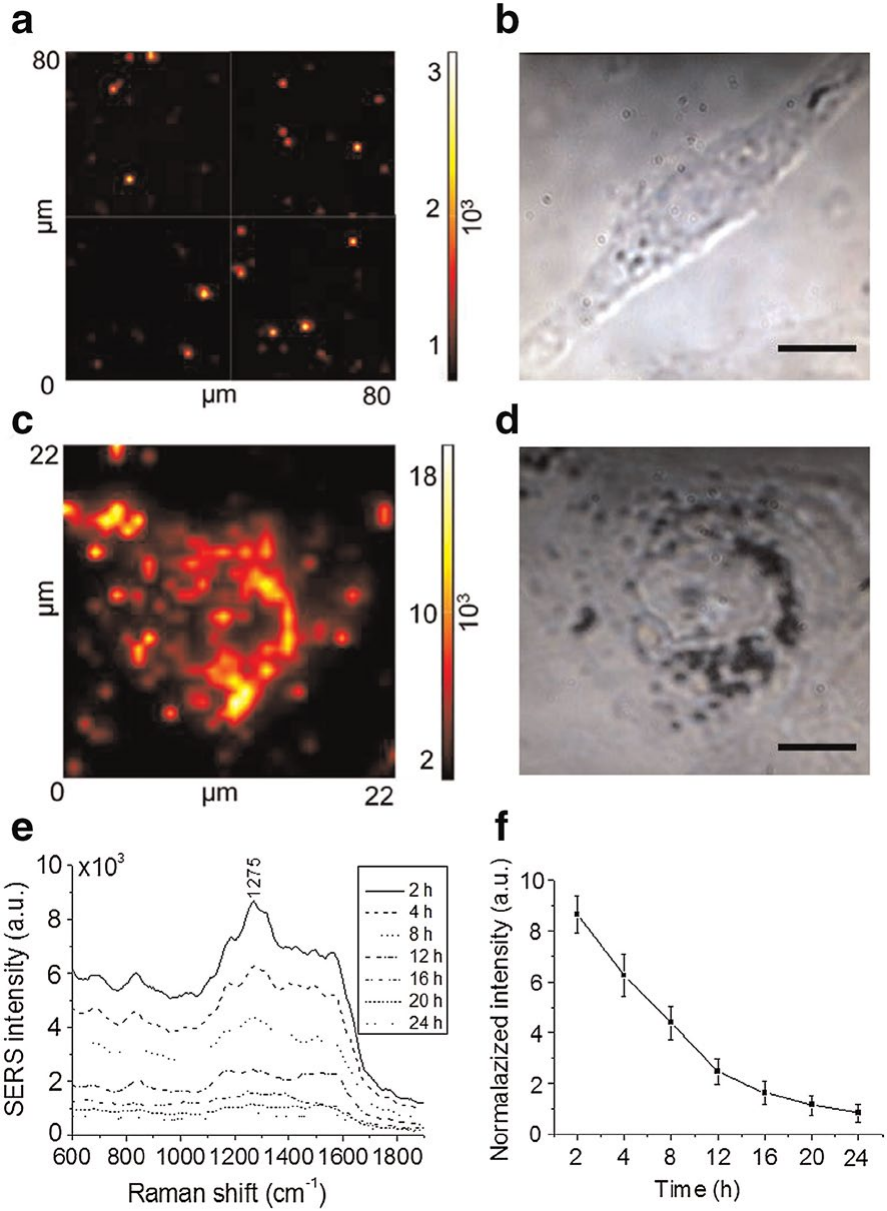
Confirmation of specific cell targeting using biohybrid nanoparticles and time dependent release of Dox inside cells. **a**, **c** SERS map images of biohybrid nanoparticles added to co-cultured SH-SY5Y and SK-BR-3 cells respectively. SERS mapping images were measured at the 1275 cm^−1^ Raman band. **b**, **d** Bright-field images of the SH-SY5Y and SK-BR-3 cells respectively. (scale bar: 20 μm (**b**), and 5 μm (**d**)). **e** SERS spectra of the SK-BR-3 cells treated with Dox loaded biohybrid nanoparticles at different incubation times. **f** Relationship between time and release of Dox. The error bars indicate the standard deviation of ten measurements in three individual cells (Reprinted with permission from [87]. Copyright @ 2015 Elsevier)

Choi’s group recently reported on the uses of SERS for monitoring the anticancer drug delivery process using Au NPs coated with Tat peptide, cell-penetrating peptide, cancer-targeting antibody, and doxorubicin (Dox) anticancer drug [87]. The Dox-loaded Au NPs showed specific SERS spectra of Dox when treated with a mixture of HER2-expressing cells (SK-BR-3) and HER2-negative cells (SH-SY5Y) [88, 89]. The cytotoxicity of the biohybrid Au NPs over 24 h treatments was investigated using MTT assay; the biohybrid Au NPs showed no cytotoxicity effect, so they could be used as a carrier for anticancer drugs, while cell treatment with Dox/Au NPs caused higher cytotoxic effects. Furthermore, Dox/Tat-C-modified Au NPs exhibited higher cytotoxic effects due to the increased uptake, and the cytotoxicity of Dox-loaded biohybrid NPs was increased as the treatment time was incremented due to the continuous release of the anticancer drug under intracellular conditions. The SERS mapping images and bright-field images of the biohybrid Au NPs–treated co-cultured SH-SY5Y and SK-BR-3 cells are, respectively shown in Fig. 5a–d, which demonstrate that the SERS signals of the biohybrid NPs were detected from SK-BR-3 cells in the immobilized area. However, low SERS signals were detected from the SH-SY5Y cell area, which indicated the specific targeting of biohybrid NPs towards cells that express HER2. The intracellular release of Dox was continuously monitored based on the changes of the intracellular SERS signal at 1275 cm^−1^ at 4-h intervals after 2 h of NPs treatment, which showed that the intracellular Dox release rate was increased in proportion with the incubation time and reached its maximum level after 12 h of NPs treatment, then the concentration and release rate decreased (Fig. 5e, f). Although the use of the targeting biohybrid NPs could enhance the specification towards the cell lines, it has a disadvantage due to the non-uniform distribution of NPs over the cell membrane, it is difficult to target the cell nucleus that is needed to cross the cell membrane and the nucleus membrane, and it could cause cytokinesis arrest, DNA damage, and result in apoptosis. In addition, the antibody was reported to cause unwanted SERS signals that could not be distinguished easily from the Raman signals that originated from target molecules inside a cell. Therefore, using modified substrates instead of metal NPs is more suitable to obtain quantitative data without cell viability.

### In vitro SERS technique to monitor the effects of different anticancer drugs based on anisotropic metal nanostructured substrates

**Fig. 6:**
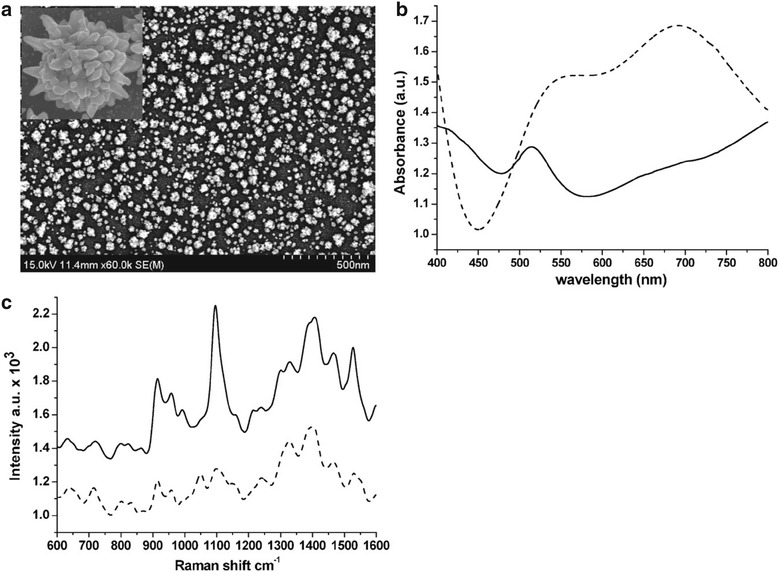
**a** SEM topography of Au nanoflower array modified ITO substrate, inset SEM image of single Au nanoflower. **b** UV–vis spectra of (solid lines) Au spherical nanoparticles/ITO and (dashed lines) Au nanoflower/ITO substrates. **c** SERS spectra of MUA immobilized on (solid lines) Au nanoflowers/ITO and (dashed lines) Au nanoparticles/ITO substrates (Reprinted with permission from [38]. Copyright @ 2010 Elsevier)

**Fig. 7:**
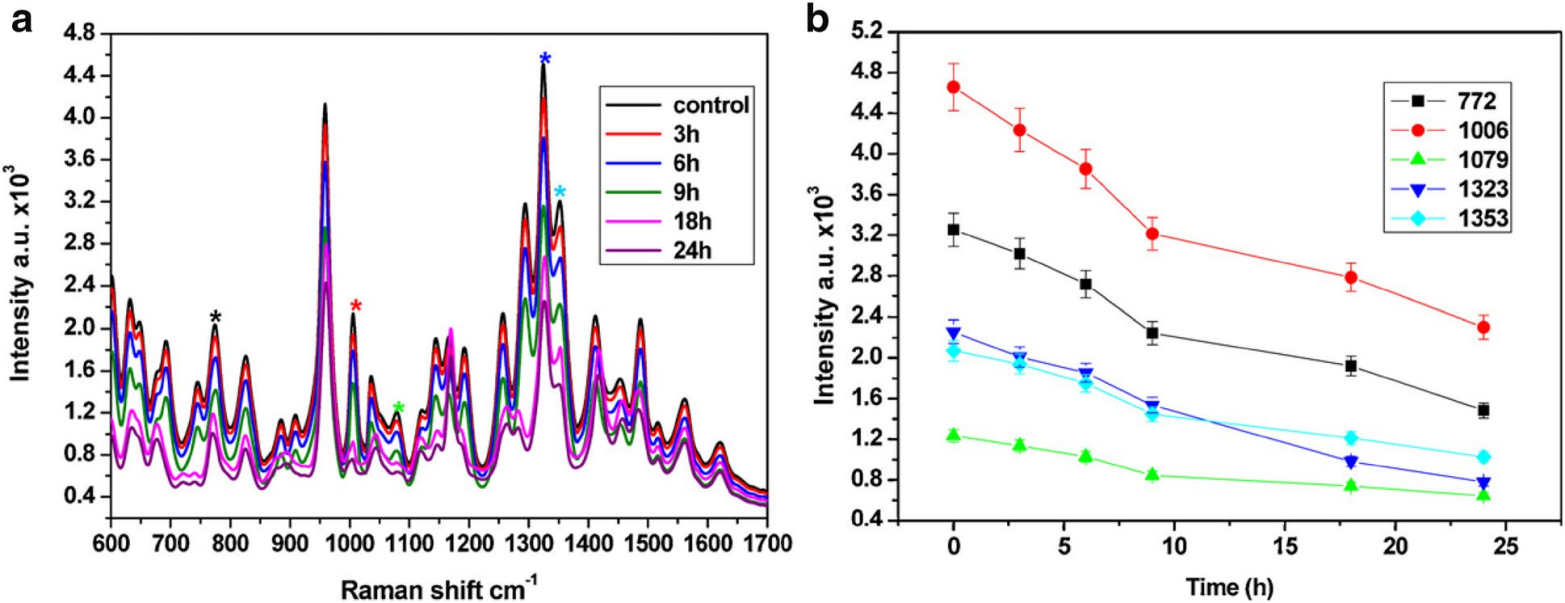
**a** SERS spectra of living HepG2 cells exposed to 1 mM of hydroxyurea, 5-fluorouracil, and cyclophosphamide, cells were treated for 24 h. **b** Changes in biochemical contents of Raman peaks from HepG2 cells treated with 1 mM of cyclophosphamide, 5-fluorouracil, and hydroxyurea. Data represent the mean ± standard deviation of ten different experiments (Reprinted with permission from [38]. Copyright @ 2010 Elsevier)

Metal anisotropic nanostructures have been reported for their enhancement of the intensity of Raman signals due to their surface plasmon resonance that amplifies the electromagnetic coupling between adsorbed molecules. El-Said et al. reported a one-step and template-free fabrication method for preparing an Au nanoflowers array-modified ITO substrate based on the electrochemical deposition of Au from an Au^3+^ solution that contains polyethylene glycol as a directing agent [38]. This substrate showed several advantages, including a broad spectral absorption band from the visible to the near-infrared, reduced fluorescence interference, minimized photo-toxicity, increased SERS effect, and penetration much deeper into the sample (Fig. 6). They have used the modified substrate to evaluate the effects of different anticancer drugs (5-FU, HU, and cyclophosphamide monohydrate) on the viability of HepG2 cancer cells based on the SERS technique. The SERS spectra of HepG2 cells after treatment with three different anticancer drugs, which indicated the cell treatment with anticancer drugs, results in the decreased intensities of SERS peaks as characterized by DNA bases (670, 748, 1323, and 1485 cm^−1^), O–P–O phosphodiester bonds (1079 cm^−1^), and that correspond to proteins (1006, 1033, 1079, and 1292 cm^−1^) compared to the SERS spectrum of the control HepG2 cell. This decrease in the amount of proteins was related to the inhibition activity of anticancer drugs for DNA synthesis, since DNA is the original template for protein synthesis. The sequential inhibition activities of anticancer drugs finally leads to cell death, which is detectable using Raman scattering due to the decrease of Raman-active species. In addition, the SERS data indicated that HU anticancer drugs showed the highest effects on the intensity of the Raman peaks corresponding to the RNAs, proteins, and DNAs of HepG2 cells due to its ability to inhibit key steps in the pathways associated with pyrimidine biosynthesis that lead to the inhibition of DNA synthesis and hence unbalanced growth and the abnormal production of mRNAs and proteins [90]. Cyclophosphamide was widely reported as an anticancer agent due to its ability to cross-link with DNA double strands that leads to the DNA backbone’s breakage [91, 92]. The SERS spectrum of HepG2 cells after treatment with cyclophosphamide, which showed a decrease in the peak intensities that corresponds to phosphate and deoxyribose, proteins and lipids confirmed the action mechanism of cyclophosphamide on cancer cells. However, the SERS spectrum of HepG2 cells treated with 5-FU showed the lowest effects on Raman peaks, particularly as the intensity of the Raman peaks decreased at 646, 1323 and 1485 cm^−1^ for DNA bases, and hence the lowest effects on cancer cell viability compared to HU or cyclophosphamide, which could be related to the specifics of the inhibition mechanism for 5-FU, since it mainly blocks the DNA and RNA synthesis of cells in the S-phase. Therefore, the cells in the other phases of the cell cycle are not affected by 5-FU compared to other drugs. The SERS spectra confirmed that the biochemical composition of several components (particularly the Raman peaks associated with DNA, RNA, proteins, and phospholipids) were changed after their treatment with anticancer drugs that completely matched previous studies of the mechanisms of anticancer drugs [93]. Furthermore, the real-time effect of HU was monitored based on analyzing the biochemical changes in living HepG2 cells during their exposure to the influence of a 200 μM solution of HU over 24 h. Figure 7 shows the decrease of Raman peaks that correspond to proteins and DNA (772 cm^−1^ peak for –O–P–O– bond and the DNA bases) (1006 cm^−1^ for phenylalanine and 1323 cm^−1^ for C–H deformation) [94, 95]. Thus, the SERS technique could be applied for the in situ monitoring of the statistical effects of drug exposure and as an effective, simple, and sensitive tool for drug screening and discovery. Furthermore, CV was used to confirm the SERS results in which the developed Au nanoflowers-modified ITO substrates could be used as a simultaneous SERS and CV substrate to screen the effects of chemotherapeutic agents on cancer cells. Although these 3D nanostructures enhance the Raman signals, the random distribution of these hot spots over the substrate results in non-uniform enhancement.

### In vitro SERS technique to to detect the differentiation between different cell lines and cell cycle stages based on a uniform metal nanostructured substrate

**Fig. 8:**
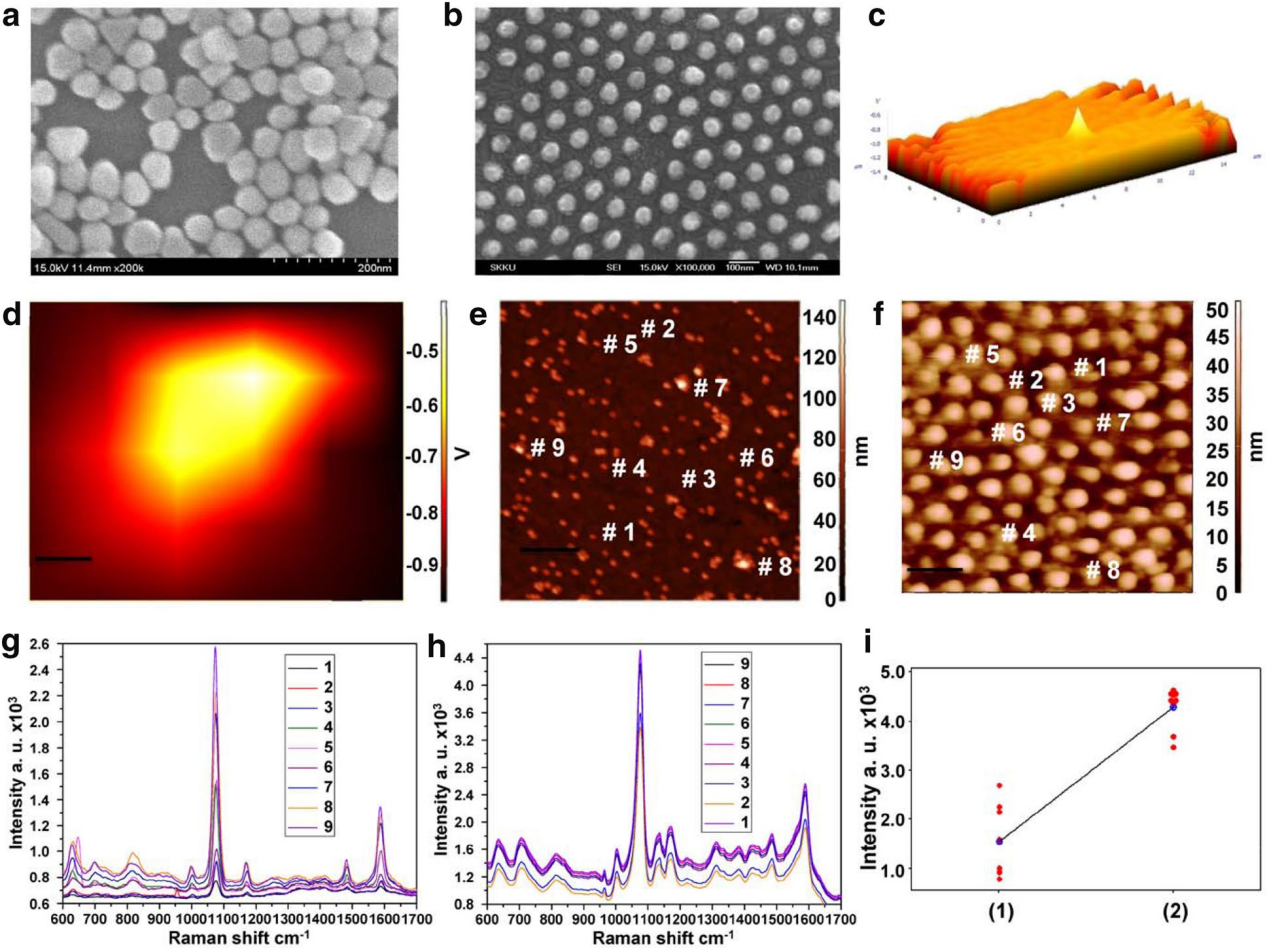
Characterization of the SERS-active substrates. **a** SEM topography of Au NPs deposited on the APTMS/ITO substrate. **b** SEM topography of the Au nanodot array fabricated on an ITO surface through an Al mask. **c** Three-dimensional confocal image of the AFM tip. **d** Confocal image of the AFM tip focused at the same point as the IR laser. **e** AFM topography of the PATP/Au NPs deposited on the APTMS/ITO substrate. **f** AFM topography of the PATP/Au nanodot array fabricated on an ITO surface. **g** SERS spectra of PATP at several points from the Au NP array substrate. **h** SERS spectra of PATP at several points from the Au nanodot array substrate. **i** Intensity distributions of the Raman peak corresponding to NH group within the SERS intensity peaks recorded from the PATP monolayer immobilized on the (1) Au NP array and (2) Au nanodot array (Reprinted from [96]. Copyright @ 2011 PloS ONE)

**Fig. 9:**
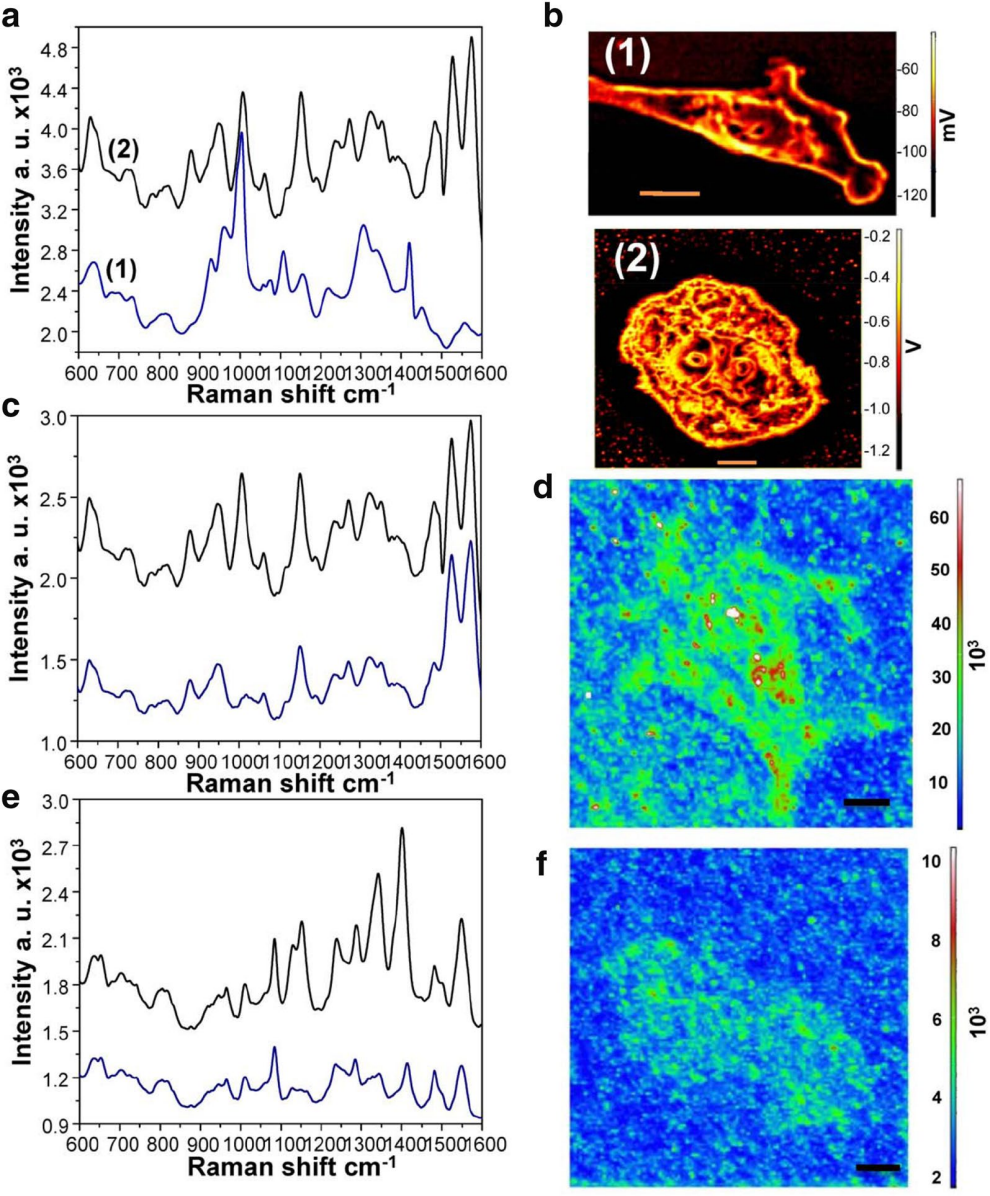
SERS monitoring at different cell cycle stages. **a** SERS spectra of (black) resting HMEC cells (G0/G1) and (blue) mitotic HMEC cells (S/G2). **b** Confocal images of HMEC cells in the (1) resting state (G0/G1) and (2) mitotic state (S/G2), **c** SERS spectra of HMEC cells from cytoplasm (blue curve) and the nucleus (black curve), and **d** SERS map image of HMEC cells. **e** SERS spectra of HepG2 cells from cytoplasm (blue curve) and the nucleus (black curve), and **f** SERS map image of HepG2 cells. Scale bar: 10 mm (Reprinted from [96]. Copyright @ 2011 PloS ONE)

El-Said et al. obtained quantitative or semi-quantitative SERS signals over a larger area and overcame the disadvantages (relatively low reliability and reproducibility) of using colloidal metal NPs or using SERS-active substrates with a random distribution of metal particles fabricated with a highly ordered SERS-active surface composed of Au nanodots/ITO substrate in which Au nanodots were thermally deposited onto the ITO surface with the assistance of a nanoporous alumina mask (Fig. 8) [96]. The highly uniform distribution of the hot spots induced Raman signal enhancement, a high signal-to-noise ratio, and low signal variances of SERS signals with high intensity and reproducibility; thus, quantitative or semi-quantitative data was obtained. The superiority of the developed uniform Au nanodots/ITO substrate was confirmed by comparison with the peak intensities of p-aminothiophenol/Au nanodots/ITO substrate with the SERS of p-aminothiophenol/non-homogeneous Au NPs/ITO; the two fabricated substrates have approximately equal numbers of Au nanodots of the same size over a large area (Fig. 8). The transparency and biocompatibility of the fabricated SERS-active Au nanodots modified surface enabled their use in several applications, including use as a cell culture system to study living cells in situ within their culture environment without any external preparation processes, for distinguishing between different cancer cell lines (MCF-7, HeLa and HepG2), between living and dead cells, between normal breast cell (HMEC) and breast cancer cell (MCF-7) lines, and can be used to distinguish cells at different cell cycle stages (G1/G0 and S/G2 phases) (Fig. 9). Furthermore, the fabricated SERS-active Au nanodots modified surface was used to differentiate the biochemical compositions of single cells at different spots, which represent the differences in biochemical compositions, enabled the differentiation of each state and the conditions of cells. This uniform SERS-active surface could be applied for single cell analysis, early cancer diagnosis, and cell physiology research.

## In vivo SERS technique using nanoparticles for cancer diagnosis

### In vivo SERS technique using nanoparticles for cancer diagnosis

Raman was applied for the in situ early detection of neoplastic lesions in the human stomach during clinical gastroscopy [97]. In addition, cancerous tissues were identified with high accuracy of about 89% and 95% inside the stomach and esophagus, respectively [97, 98]. Raman could be used for the ex vivo diagnosis of human breast cancer with high sensitivity (83%) and specificity (93%) [99]. The sensitivity and specificity of Raman spectroscopy for the in situ diagnosis of colorectal cancer in single live epithelial cells were both approximately 86.3% [100].

**Fig. 10:**
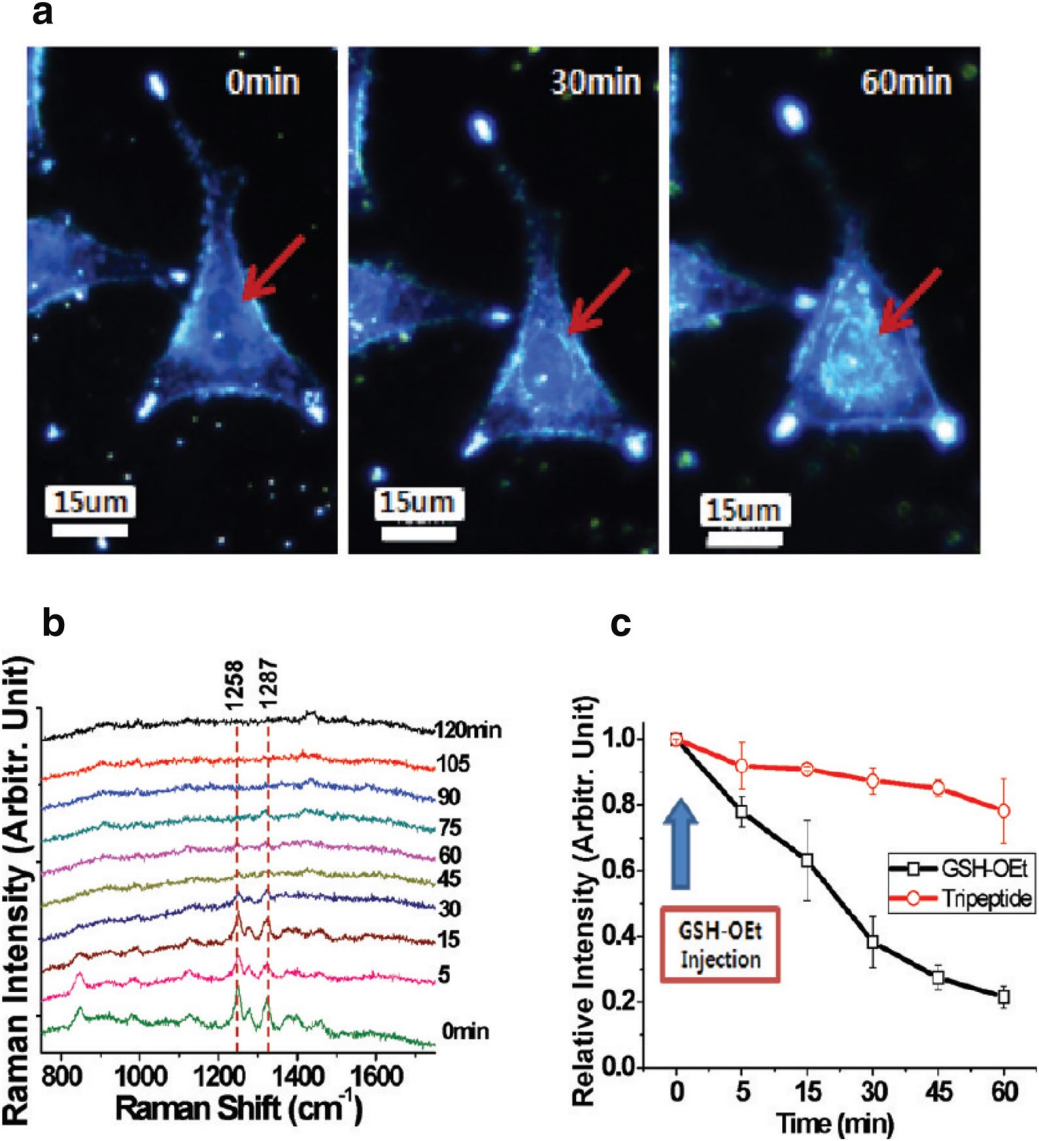
**a** Time-lapse DFM live cell images in a single A549 cell after treatment of glutathione ethyl ester (GSH-OEt) indicating the in situ release of 6MP from Au NPs. The arrow indicates the position where the Raman spectra were obtained. The Au NPs were incubated for 24 h prior to the GSH-OEt treatment. **b** The peak at 1258 cm^−1^ was monitored with time. The other vibrational bands of 6MP also showed similar behaviors. Raman spectra were obtained after a few minutes from the injection time of GSH-OEt. **c** Plot of decrease in the band intensities depending on the elapsed time after GSH-OEt injection. The error bar indicated the standard deviation of the three measurements. GSH-OEt and tripeptides were marked in black and red, respectively (Reprinted with permission from [101]. Copyright @ 2002 American Chemical Society)

**Fig. 11:**
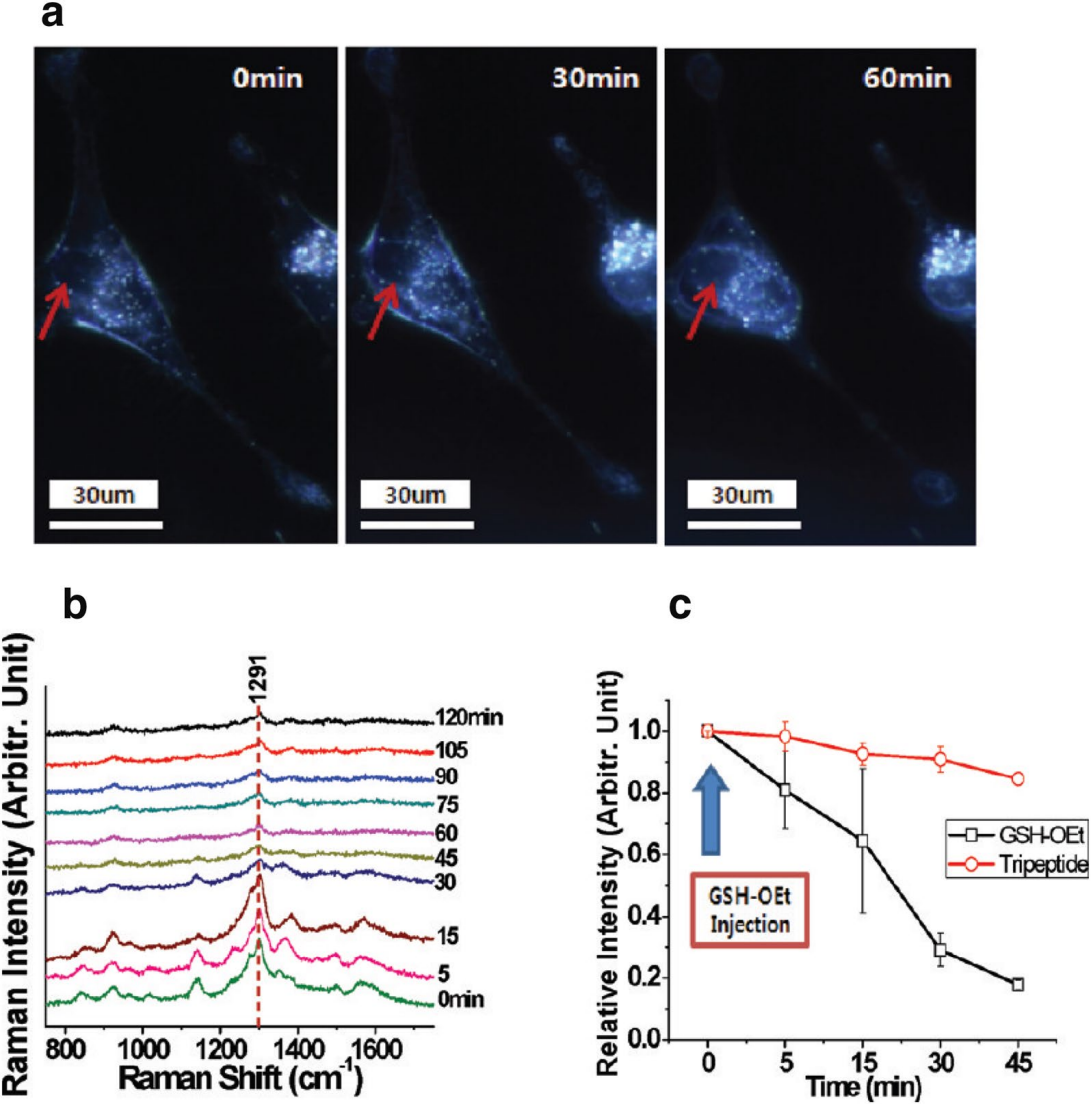
**a** Time-lapse DFM live cell images in a single HeLa cell after treatment of glutathione ethyl ester (GSH-OEt) indicating the in situ release of 6TG from Au NPs. The arrow indicates the position where the Raman spectra were obtained. **b** The strongest peak at 1291 cm^−1^ from 6TG was used. **c** Plot of decrease in the band intensities depending on the elapsed time after GSH-OEt injection (Reprinted with permission from [101]. Copyright @ 2002 American Chemical Society)

Ock et al. reported the use of confocal Raman spectroscopy as a label-free and real-time tool for the in vitro and in vivo investigation of glutathione (GSH)-induced intracellular thiopurine anticancer drug release of 6-mercaptopurine (6MP) and 6-thioguanine (6TG) adsorbed on Au NPs, in which glutathione monoester (GSH-OEt) was used as an intracellular external stimulus to trigger the drug release [101]. The cellular uptake of the drug/Au NPs was confirmed using TEM and fluorescence images, with confirmation of the intracellular distribution of Au NPs in both A549 and K562 cells that was mainly presented as an aggregated form inside the vesicular structures, but TEM and fluorescence images only represent static information. Thus, SERS and Dark-field microscopy (DFM) techniques were used for the real-time monitoring of the uptake and release of 6MP and 6TG from the drug-modified Au NPs surfaces. The DFM images and SERS spectra have confirmed the uptake of the drug-modified Au NPs inside the A549 cell. Real-time DFM imaging for the release of 6MP and 6TG into the A549 live cell were, respectively represented in Figs. 10, 11, which indicate the release of thiopurine drugs after the injection of GSH inside the cell with sufficient elapsed time. Furthermore, the SERS spectra showed a significant decrease in the SERS intensities (about 40–70%) after the injection of GSH-OEt into the cell, which confirmed the drug’s release from the Au NPs’ surfaces. The SERS data showed the capability to monitor the release of very low concentrations of 6MP and 6TG drugs after the injection of GSH that reached the nanomolar range (about 1.8 × 10^−9^ M of 6MP), which was lower than the IC_50_ values of 6MP and 6TG (micromolar range) [102, 103]. However, one limitation of this strategy was the aggregation of Au NPs inside cells that could affect the SERS intensity and induce unequal enhancement, the obtained results could thus be considered qualitative data. In addition, the SERS effect depends on the adsorbate molecules, the geometry of nanostructures including hot spots, and the nature of the nanostructures. These in vitro data were also confirmed using fluorescence images; they have also mentioned the use of SERS for the in vivo monitoring of the release of 6TG anticancer drug from Cy5-dye assembled on 6TG-capped Au NPs in living mice after injection with GSH. The in vivo SERS spectra of 6TG exhibit a Raman peak at 1291 cm^−1^, which was a characteristic peak of 6TG, and this peak’s intensity was decreased after injection with GSH-OEt compared to the control. In addition, they have reported that 6MP-coated Au NPs did not cause any acute toxicity but reduced the tumor volume after intratumoral injection to the HeLa cells xenografted to nude mice after a 2-week treatment period, which indicated the capability of the SERS technique for the in vitro and in vivo screening of the distribution of anticancer drugs. Colorectal cancer is the third most common cancer in men and women [104] and it is difficult to determine whether a tumor is malignant or benign based solely on its visual appearance [105, 106]. Therefore, developing a technique for the in situ analysis of the molecular compositions of adenomas and adenocarcinomas and for monitoring the effect of anticancer drugs is urgently important. Taketani et al. applied a miniaturized Raman endoscope (mRE) system as a noninvasive in situ method for monitoring the advancement of colorectal tumors in model mice and to visualize and measure the Raman spectra of any targeted point within the colorectal tumor without damaging the tissues [107]. The Raman spectra of the live tumor model and of controlled mice were measured from several polyp-like tumors and from the control at different ages of up to 19 weeks. The Raman spectra of the tumor showed several peaks at 1662, 1451, and 1342 cm^−1^, which were characteristics for the vibrational of amide I, C–H bending, and amide III of protein, respectively. It seemed that the Raman peak at 1003 cm^−1^ was assigned to the breathing mode of the phenyl ring of phenylalanine [108], while the Raman peak at 933 cm^−1^ can be assigned to proline in collagen type I [109, 110]. In addition, the Raman peaks at 1736, 1655, and 1441 cm^−1^ were assigned for the bending of C=O stretching, C=C stretching, and C–H groups of triacylglycerol, respectively [111, 112]. The Raman spectra showed efficient performance for the discrimination of normal tissues from tumor tissues with 86.8% accuracy. These studies showed the possibility of applying Raman spectroscopy for the in vivo monitoring of the unique changes in the molecular composition of the tumor, and for studying the therapeutic effects of anticancer drugs and other medical treatments that could reduce the number of mice to be killed.

### In vivo SERS technique using nanoparticles to monitor the drug distribution inside a cell and the cell’s metabolism

**Fig. 12:**
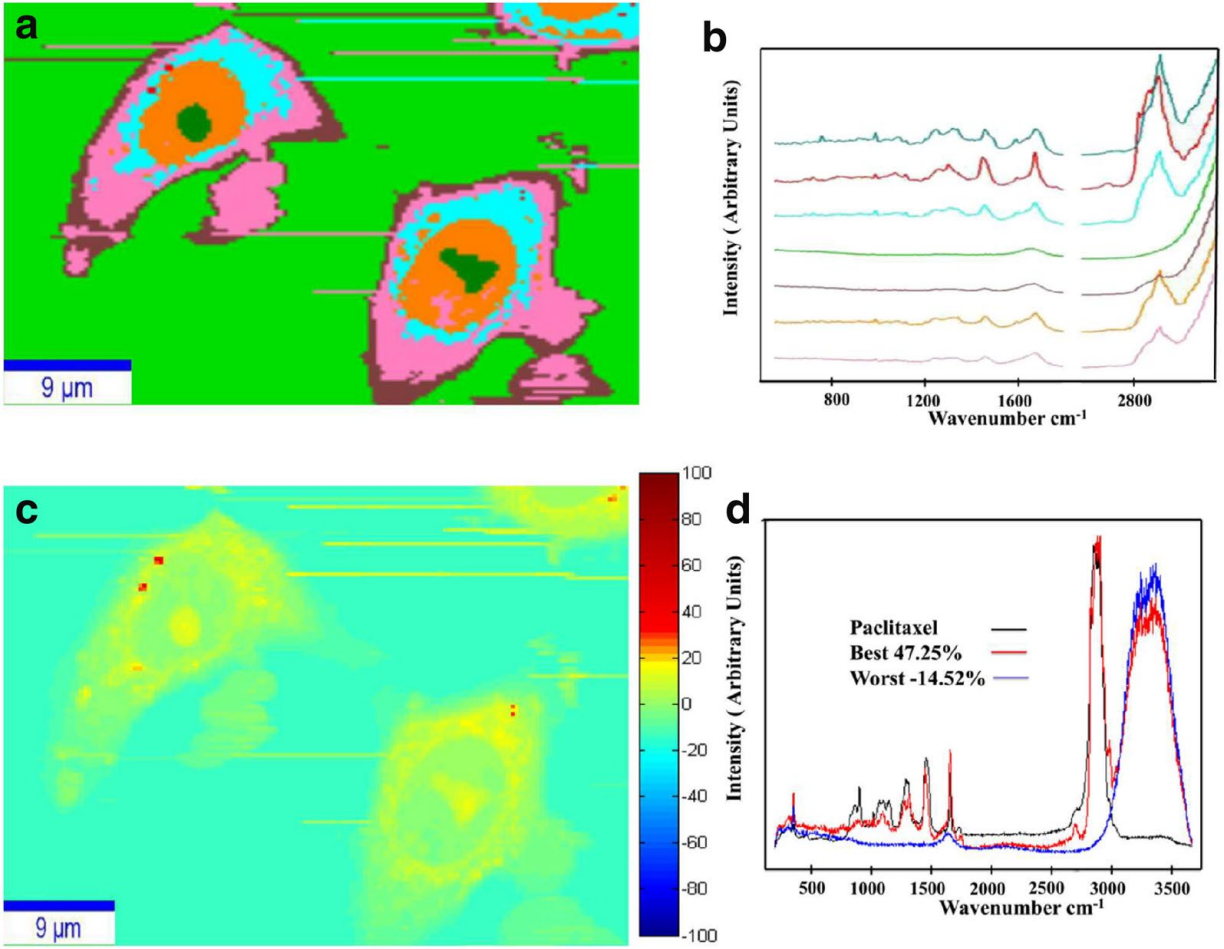
**a** Seven cluster Raman map of MCF-7 cells. **b** Average spectra corresponding to clusters in (**a**) (same colors as in (**a**)): the average spectra of nucleus (orange) nucleolus (dark green), cytoplasm (pink), membrane (brown), endoplasmic reticulum (light blue), PBS buffer (light green) and paclitaxel (red). **c** Correlation map of the same image. **d** The correlation coefficient between the whole spectra and the one of paclitaxel taken as a reference (black spectrum). The best correlation is obtained for the paclitaxel in cell (red spectrum); the region with no correlation to paclitaxel is due to the PBS buffer (blue spectrum) (Reprinted with permission from [120]. Copyright @ 2013 AIP)

**Fig. 13:**
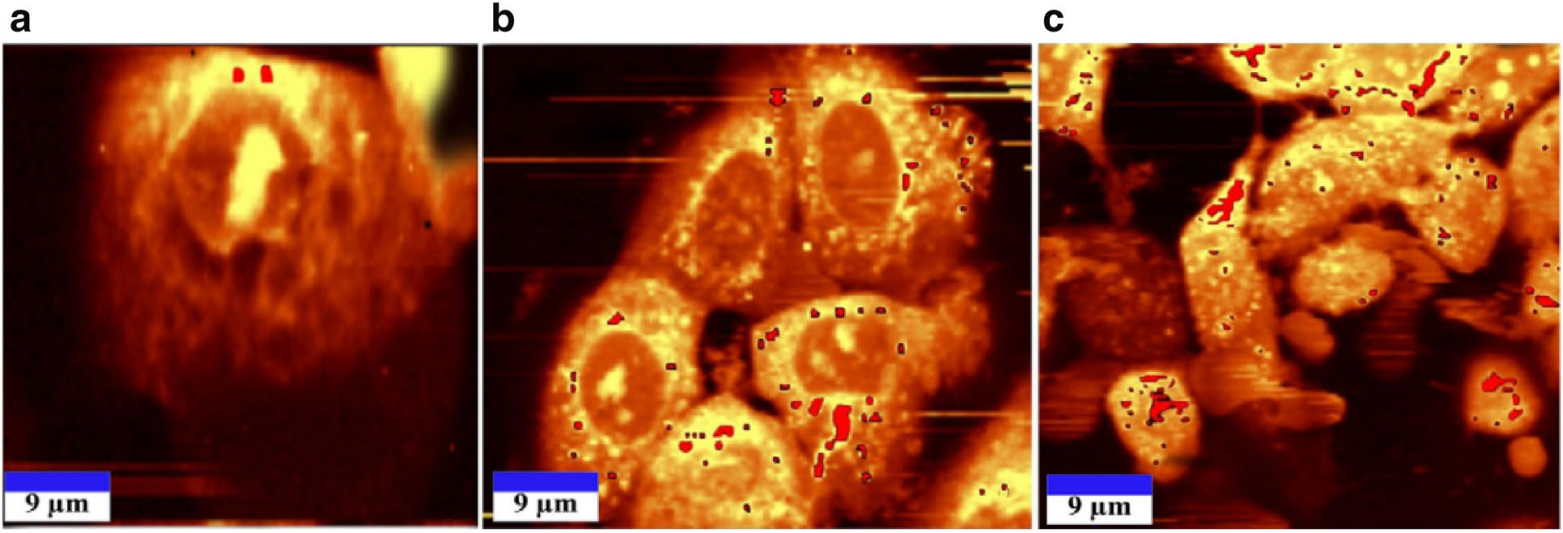
Raman images of MCF-7 cells incubated in paclitaxel. Integrated Raman intensities in the 2800–3000 cm^−1^ region of the cell, marking paclitaxel as red spot. **a** 3 h; **b** 6 h; **c** 9 h incubation of cells in culture medium containing paclitaxel. Reprinted with permission from [120]. Copyright @ 2013 AIP

The identification of the metabolism and distribution of different chemotherapy drugs inside a single cell is an important issue in patient management. Fluorescence techniques have been established for studying the distribution and mapping of drugs in fixed and live cells [113], but it has some drawbacks such as low contrast and photo-bleaching. It needed to introduce fluorescent labels that could alter the biochemical properties of a molecule of interest, and many anticancer drugs are non-fluorescent. Raman microscopy was used as a label-free tool to trace drugs within cells, image individual cells, perform protein conformation imaging, and localize intracellular nanoparticles [113–118]. Here, we will discuss the applications of Raman spectroscopy to monitor the distribution of non-fluorescent drugs within living cells. Salehi et al. [119] used a Raman imaging technique to study the distribution of traces of a paclitaxel anticancer drug inside living MCF-7 cells based on monitoring the changes in the total integrated Raman intensities of the C=O stretching band (1740 cm^−1^) that characterize paclitaxel in cells. Figure 12c depicts a Raman image of treated MCF-7 cells, which revealed several clusters that correspond to different cellular components including the nucleus (orange) nucleolus (dark green), cytoplasm (pink), membrane (brown), endoplasmic reticulum (light blue), and paclitaxel (red). The distribution of paclitaxel inside MCF-7 cells can be studied using Raman images of MCF-7 cells after different treatment periods with paclitaxel solution, as represented in Fig. 13, which indicates that paclitaxel passes the cell membrane and enters the cytoplasm and then gradually diffuses into the cytoplasm without entering the cell nucleus. Moreover, Draux et al. [120] used a Raman map to evaluate the effect of different doses and incubation times of gemcitabine as antitumor drug on a single living Calu-1 cell based on monitoring the changes in the Raman signals from biomolecules (DNA, RNA, and proteins) of the control and treated cells. Although these studies showed the ability to monitor the drug distribution inside cells, they did not show any information about how the drug is metabolized.

**Fig. 14:**
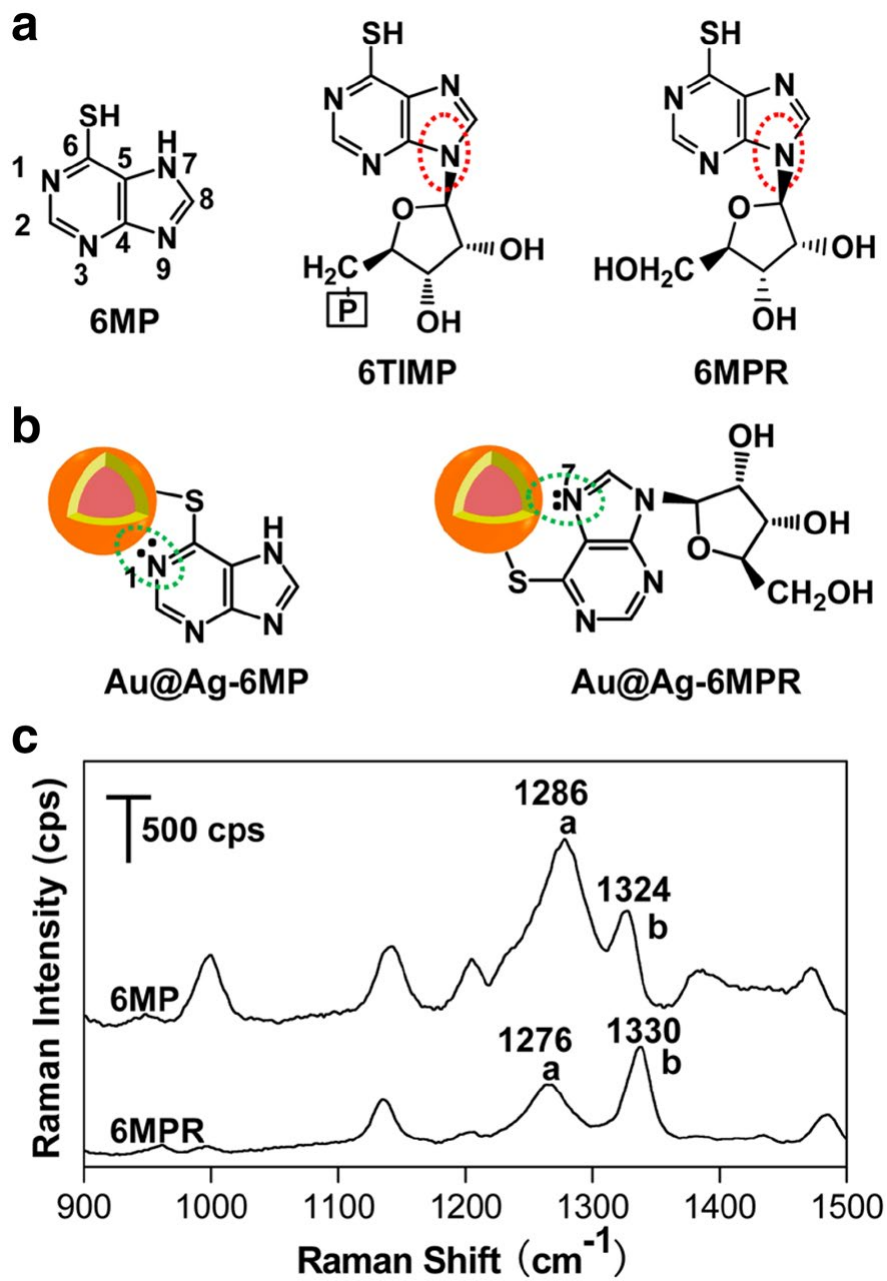
**a** Chemical structures of 6MP and its riboside derivatives 6TIMP and 6MPR (N9 positions were indicated with the red circles). **b** The different adsorption behaviors of 6MP and 6MPR on the surface of Au@Ag NPs. **c** SERS spectra of 6MP and 6MPR (1 × 10^−6^ M) in the colloid of Au@Ag NPs. All the Raman spectra were recorded with 532 nm laser (Reprinted with permission from [124]. Copyright @ 2014 American Chemical Society)

**Fig. 15:**
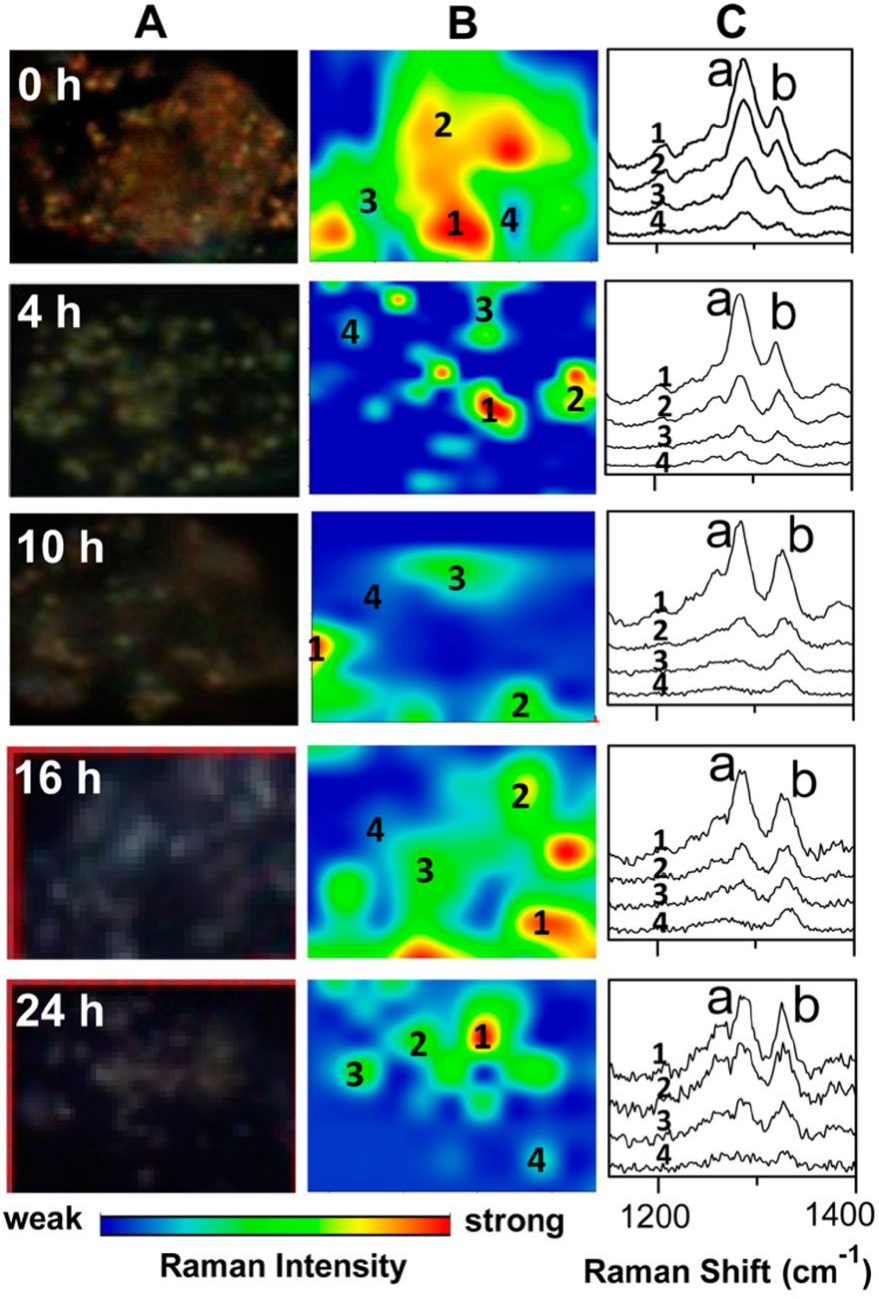
SERS mapping to monitor the metabolism of 6MP in individual A549 cells over time (the uptake of Au@Ag-6MP by the incubation for 5 h). **A** Dark-field optical images of Au@Ag-6MPuploaded cells with different incubation times in fresh culture medium (0, 4, 10, 16, and 24 h). **B** SERS images mapped with the Raman bands at 1286 cm^−1^ (0 h) and 1330 cm^−1^ (4, 10, 16, and 24 h). **C** The corresponding Raman spectra at the indicated sites in the left Raman images. (The color bar indicates the Raman signal intensity) (Reprinted with permission from [124]. Copyright @ 2014 American Chemical Society)

6-Mercaptopurine (6MP) is a purine derivative anticancer drug. 6MP itself is anticancer-inactive, but it can be converted via an enzymatic reaction into anticancer-active intermediates (first to 6-thioinosine monophosphate (6TIMP) and then to 6-mercaptopurine-ribose (6MPR)) as shown in Fig. 14a. 6MPR was reportedly incorporated into DNA and RNA sequences and thus prevented the replication of cancer cells (Fig. 14a) [121]. Yang et al. [122] reported the simultaneous monitoring of 6MP and methimazole (MMI) drugs inside living HeLa cells based on the SERS technique. They investigated the intracellular diffusion and metabolism of MMI and 6MP drugs and mentioned that the metabolism rate of 6MP in living HeLa cells is much faster than that of MMI. These results indicated the SERS technique’s capability for real-time simultaneous monitoring of a mixture of different drugs inside a single living HeLa cell. Han et al. used the SERS technique by using Au/Ag NPs core/shell NPs for real-time imaging and monitoring the metabolism and to understand the molecular therapeutic mechanism of 6MP as an antitumor drug in A549 living cells at the cellular level [123]. The Raman spectra of 1 μM of 6MP and 6MPR in the colloid of Au/Ag NPs showed almost the same fingerprints that mainly come from the purine ring and represent two main peaks at a 1324 cm^−1^ and b 1286 cm^−1^. However, the ratio of the intensity of b to a (Ib/Ia) in 6MP was less than in 6MPR (Fig. 14c), which indicates that the structure and adsorption of 6MP and 6MPR on Au/Ag NPs was different. The detection limit of 6MP and 6MPR was 10 nM using Au/Ag NPs, which was lower than the clinical treatment concentrations of 6MP (1–100 μM). The distribution of Au/Ag-6MP NPs inside A549 cells was confirmed using dark-field optical images (Fig. 15, column A), which revealed that the A549 cells had absorbed a large amount of Au/Ag-6MP NPs at 0 h. The metabolism of 6MP adsorbed on Au/Ag NPs at four different regions in the cells was monitored at different treatment times over 24 h using Raman images. At 0 h, the distribution of Au/Ag-6MP in A549 cells was investigated by the Raman image for a Raman peak at 1286 cm^−1^. While the metabolism of 6MP into 6MPR at 4, 10, 16, and 24 h of cell treatment was studied by the Raman image for the Raman peak at 1330 cm^−1^ (Fig. 15, column B), which indicated the increase of the Ib/Ia ratio at the four different sites in the cells with increasing treatment time. In addition, they mentioned that the rate of metabolism depends on the density of Au/Ag-6MP in the cells; the sites that contain a large amount of Au/Ag-6MP showed a lower biological conversion rate of 6MP into 6MPR compared to sites that have less Au/Ag-6MP due to the aggregation of Au/Ag-6MP, which reduced the efficiency of enzymes and riboses toward Au/Ag-6MP. Furthermore, they suggested that 6MPR might undergo further biological conversions or take part in the synthesis of DNA/RNA. These results illustrated the capability of Raman imaging for the real-time visualization of the distribution of 6MP and its metabolism in tumor cells. In addition, Gerwert’s group used Raman microscopy to follow the uptake of the molecular targeted agent erlotinib, an anticancer drug, to the tyrosine kinase domain of EGFR within colon cancer cells (SW480) [124]. Erlotinib, which has different side-chains, was reported to undergo extensive enzymatic conversion into its metabolism through three main paths: (i) O-demethylation of the side chain that could be oxidized into the carboxylic acid form, (ii) oxidation of the acetylene moiety to aryl carboxylic acid, and (iii) 4-hydroxylation of the phenyl-acetylene moiety (IV) [125, 126]. In in vitro studies, erlotinib was mixed with captisol as a drug carrier [127]; in another in vivo study, erlotinib was given orally in combination with captisol. The Raman images of SW480 cells after treatment with erlotinib for 12 h indicated that erlotinib was distributed in the cell periphery and clustered at the EGFR protein in the cell membrane. Monitoring the changes of the Raman spectrum for SW480 cells before and after treatment with erlotinib demonstrated that it was metabolized into its demethylated derivative, which has been reported previously based on different chromatography techniques [128–130]. These studies indicated that Raman microscopy could be used as a simple, non-invasive, label-free, and efficient technique for assessing drug efficacy to investigate pharmacokinetics at the highest possible resolution in living cells and understand the molecular therapeutic mechanisms of antitumor drugs at the single-cell level.

## Conclusions

Electrochemical techniques such as electrochemical cell chips and optical techniques such as SERS based on a nanostructured surface have shown high capacity to evaluate the efficiency of anticancer drugs. Electrochemical cell-based chips are quick, easy, and more sensitive than optical methods for differentiation between cancer and healthy cell lines, for assessing cell viability, and for determining the efficiency of several kinds of anticancer drugs and environmental toxins. Modified carbon electrodes have been used to study cell viability based on their electrochemical responses that demonstrate irreversible behavior, and the oxidation peak disappeared in the second scan cycle. This indicated instability in the electrochemical response of cells at the modified carbon electrodes and could hence result in inaccurate results. However, since nanostructured Au electrodes showed a quasi-reversible and stable response for different cell lines, the use of nanostructured Au surfaces for developing electrochemical cell-based chips has shown superior performance for cancer diagnosis and the assessment of cell viability. SERS techniques based on nanopatterned surfaces have been applied to monitor the cell metabolism related to anticancer drugs. Nanostructured Au substrates could be used for simultaneous detection by SERS and CV to monitor the effects of chemotherapeutic agents on cancer cells. Spectroelectrochemical techniques based on nanostructured surfaces led to the development of a simple, non-invasive, label-free, and efficient technique for the assessment of drug efficacy at the highest possible resolution in living cells and to develop the understanding of the molecular therapeutic mechanism of antitumor drugs at the single-cell level. Thus, uses of nanostructured surface for developing electrochemical and SERS-based chips have been reviewed. We can conclude that electrochemical cell-based chips are useful, simple, and highly sensitive candidates for evolving the in vitro efficiency of different anticancer drugs. However, the uses of uniform nanostructured surfaces as SERS-active substrates for developing cell-based chips could be considered an excellent nondestructive bioanalytical tool for monitoring the efficiency of anticancer drugs and for the real-time studying of action mechanism of different anticancer drugs for both in vitro and in vivo studies. Furthermore, the use of nanostructured surfaces avoids the side effects of nanostructured colloidal solution that could induce damage in the cell membrane.
